# Long‐term outcomes of active surveillance for low‐risk papillary thyroid carcinoma: Progression patterns and tumor calcification

**DOI:** 10.1002/wjs.12417

**Published:** 2024-11-26

**Authors:** Iwao Sugitani, Ryuta Nagaoka, Marie Saitou, Masaomi Sen, Hiroko Kazusaka, Mami Matsui, Takeshi Abe, Ryo Ito, Kazuhisa Toda

**Affiliations:** ^1^ Department of Endocrine Surgery Nippon Medical School Tokyo Japan; ^2^ Division of Head and Neck Cancer Institute Hospital Tokyo Japan

**Keywords:** endocrine, head and neck, oncology, outcomes

## Abstract

**Introduction:**

Active surveillance (AS) for low‐risk papillary thyroid carcinoma (PTC) is acknowledged as a valid management strategy. While older age is identified as a favorable factor for progression, long‐term evidence is scarce and lifelong monitoring has been deemed essential. This study investigated progression patterns and tumor calcification under long‐term AS and explored the possibility of ending follow‐up.

**Materials and Methods:**

A total of 650 patients with low‐risk PTC who chose AS were enrolled. Progression was defined as either tumor enlargement (≥3 mm from initiation) or development of clinically apparent lymph node metastasis.

**Results:**

The median observation period was 8 years; 45.2% were under surveillance for ≥10 years. Overall, 80 patients (12.3%) exhibited progression. Median age and observation period at the time of progression were 55 and 4 years, respectively. Only 2 patients showed progression after 15 years of follow‐up and 5 patients showed progression after reaching 80 years old. Among 71 patients experiencing tumor enlargement, surgery was performed immediately in 32 patients. The remaining 39 patients continued surveillance, but only 5 demonstrated ongoing enlargement thereafter. Of 40 surgeries due to progression, 36 were conducted within the first 10 years. The degree of calcification correlated with age and observation periods. No progression occurred after the development of rim calcification.

**Conclusions:**

Progression during AS was extremely rare in older patients with long‐term surveillance and in tumors with rim calcification. It may be feasible to consider ending scheduled surveillance visits for these patients. Instances of progression halting after enlargement are not uncommon.

## Graphical abstract

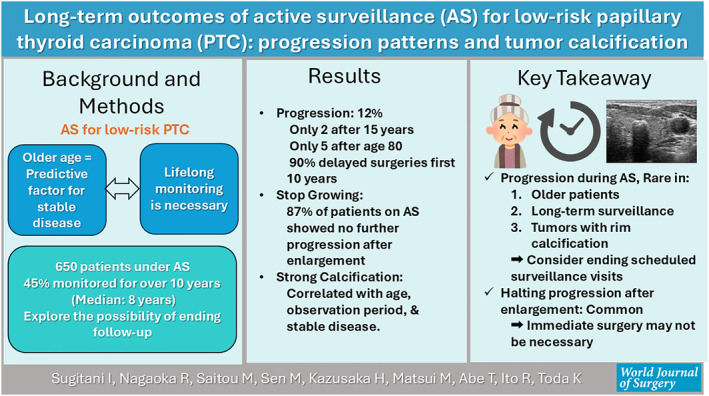

## 1 INTRODUCTION

In recent years, the incidental detection of small papillary thyroid carcinoma (PTC) has been increasing in various developed countries.^1^, ^2^ This increase is mainly attributed to the widespread use of ultrasonography (US) and indeed the mortality of thyroid carcinoma has not changed. To reduce overtreatment of low‐risk PTC, prospective clinical trials have been investigating active surveillance (AS) for patients with cT1aN0M0 PTC at two Japanese institutions since the 1990s.^3^, ^4^ Based on the favorable outcomes of these trials, AS has been gradually adopted worldwide as a valid alternative to immediate surgery.^5^, ^6^

The accumulation of studies on AS for low‐risk PTC has provided various insights into the natural history of this pathology. Age has been identified as a significant predictor of progression under AS,^7^, ^8^ and tumors initially showing some degree of enlargement often undergo subsequent cessation of progression.^9^ In addition, although weak tumor calcification and rich tumor vascularity are associated with greater likelihoods of enlargement, calcification tends to intensify and tumor vascularity is prone to decrease during AS.^10^

While older individuals exhibit lower probabilities of progression, old age is regarded as one of the most important predictors of poor prognosis for thyroid cancer in general.^11^ Consensus statements from the Japan Association of Endocrine Surgery (JAES)^12^ have recommended lifelong monitoring with AS. At present, no long‐term evidence is available regarding after how many years or at what age AS can be terminated. This study aimed to examine progression and calcification patterns in low‐risk PTC under long‐term AS since 1995, assessing the feasibility of de‐escalating surveillance.

## 2 MATERIALS AND METHODS

### 2.1 Study design and patients

This study was conducted as a retrospective analysis of prospectively collected data. A prospective clinical trial of AS for patients with low‐risk papillary thyroid microcarcinoma (PTMC) has been conducted since 1995 at Cancer Institute Hospital^4^ and the same protocol has been provided as a management option in daily medical practice since 2013 at Nippon Medical School Hospital.^13^ All patients with PTC diagnosed by US‐guided fine needle aspiration cytology were evaluated for the presence of clinically apparent lymphadenopathy (maximum diameter ≥1 cm), extrathyroidal extension, and distant metastasis using neck US, computed tomography of the chest, and laryngoscopy. For all patients with cT1aN0M0 PTC and some patients who specifically requested AS for cT1bN0M0 PTC (usually with maximum diameter <15 mm^14^), detailed information was provided regarding the option of AS in addition to immediate surgery using the same handout at both institutions. Each patient then autonomously selected their preferred management option. Eventually, 650 patients (554 patients with T1a and 96 patients with T1b) who chose AS during the period between 1995 and 2022 were enrolled. Patients with at least 1 year of follow‐up were included.

### 2.2 Protocol of AS

When a patient chose AS, the tumor was surveyed every six or 12 months in accordance with the previously described protocol.^12^, ^13^ Progression was defined as either tumor enlargement or development of clinically apparent lymph node metastasis (LNM). Enlargement or reduction of tumor size was defined as a change in maximal tumor diameter ≥3 mm on US from the start of observation according to the JAES consensus statements.^12^ Development of LNM was diagnosed mainly by US. Conversion surgery was performed when a patient changed his/her preference, concerns arose regarding extrathyroidal extension to the recurrent laryngeal nerve, trachea, or esophagus, LNM appeared, or tumor diameter reached 13 mm in general.

### 2.3 Definition of tumor calcification patterns

**FIGURE 1:**
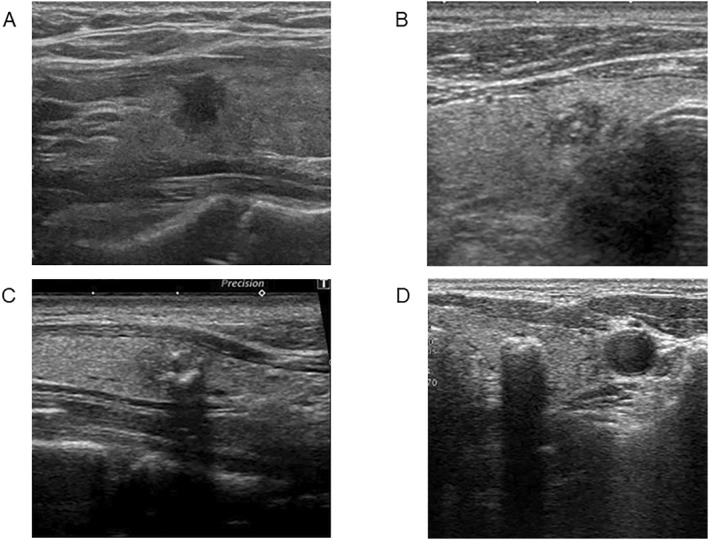
Tumor calcification patterns on ultrasonography. (A) No calcification. 46 year‐old female, sagittal section of the left thyroid lobe. (B) Microcalcification: 76 year‐old male, transverse section of the right thyroid lobe. (C) Macrocalcification: 51 year‐old female, sagittal section of the left thyroid lobe. (D) Rim calcification: 78 year‐old female, transverse section of the left thyroid lobe.

According to the US findings, tumor calcification was classified into the following four patterns: (a) no calcification, indicating an absence of any calcification within the tumor; (b) microcalcification, characterized by single or multiple small (<1 mm) spots of calcification without acoustic shadows; (c) macrocalcification, consisting of relatively large (≥1 mm) or clustered calcification causing acoustic shadows; and (d) rim calcification, with calcification aligned along the tumor rim, resulting in a complete acoustic shadow (Figure 1). These patterns were broadly grouped into “weak calcification” for no calcification or microcalcification and “strong calcification” for macrocalcification or rim calcification.^10^

### 2.4 Definition of tumor enlargement patterns

Patterns of change in tumor size were categorized into the following five groups: (a) stable, encompassing cases not fitting any of the other criteria; (b) early enlargement, defined as an increase of ≥3 mm in maximal tumor diameter with ≤5 years of AS; (c) late enlargement, characterized by an increase of ≥3 mm reached with >5 years of AS; (d) late but rapid enlargement, representing cases experiencing initial stability for >5 years followed by an increase of ≥3 mm in ≤1 year; and (e) reduction, indicating a decrease of ≥3 mm. Furthermore, groups II and III were further divided into cases in which enlargement continued thereafter (≥2 mm increase over a period of ≥3 years) and cases in which enlargement ceased. However, cases with an observation period of <3 years after the confirmation of enlargement were classified as indeterminate.

### 2.5 Statistical analysis

Comparisons between groups were performed using Fisher's exact test for categorical data and the Kruskal–Wallis test for continuous variables. We employed Kaplan–Meier survival analysis to estimate time‐to‐event data for key outcomes such as progression‐free survival and conversion surgery‐free survival. Multivariate analysis for predictors of progression was carried out by Cox proportional hazard modeling incorporating factors that had been validated as potential prognostic factors for low‐risk PTC under AS.^4^, ^7^, ^13^, ^15^ All analyses were performed using EZR (Saitama Medical Center, Jichi Medical University, Saitama, Japan).^16^ Values of *p* < 0.05 were considered statistically significant.

## 3 RESULTS

Clinical characteristics for the entire cohort are shown in Table 1. Mean age at presentation was 53.4 ± 12.8 years and median duration of follow‐up was 8 years, with 294 cases (45.2%) under surveillance for ≥10 years.

**TABLE 1:** Characteristics of patients with low‐risk papillary thyroid carcinoma who underwent active surveillance.

Characteristics	*N* = 650
Sex (female/male)	557 (85.7%)/93 (14.3%)
Initial age (years)	Mean, 53.4 ± 12.8; median, 54 (range, 15–86)
Age at last exam (years)	Mean, 62.3 ± 14.1; median, 63 (range, 21–95)
Duration of follow‐up (years)	Mean, 8.9 ± 5.7; median, 8 (range, 1–29)
Duration of follow‐up (<10 years/≥10 years)	356 (54.8%)/294 (45.2%)
Initial tumor size (mm)	Mean, 8.2 ± 2.2; median, 8 (range, 2–17)
T stage (T1a/T1b)	554 (85.2%)/96 (14.8%)
Initial TSH (mIU/L)	Mean, 1.97 ± 1.92; median, 1.65 (range, 0.01–26.8)
TgAb and/or TPOAb (either positive/both negative)	425 (65.4%)/225 (34.6%)
Multifocality (absent/present)	516 (79.4%)/134 (20.6%)
Family history of thyroid cancer (absent/present)	629 (96.8%)/21 (3.2%)
History of other malignancy (absent/present)	503 (77.4%)/147 (22.6%)
Initial tumor calcification	
No calcification	148 (22.8%)
Microcalcification	316 (48.6%)
Macrocalcification	148 (22.8%)
Rim calcification	38 (5.8%)
Initial tumor vascularity (poor/rich)	555 (85.4%)/95 (14.6%)

*Note*: TSH, thyrotropin; TgAb, anti‐thyroglobulin antibody; TPOAb, anti‐thyroid peroxidase antibody.

**FIGURE 2:**
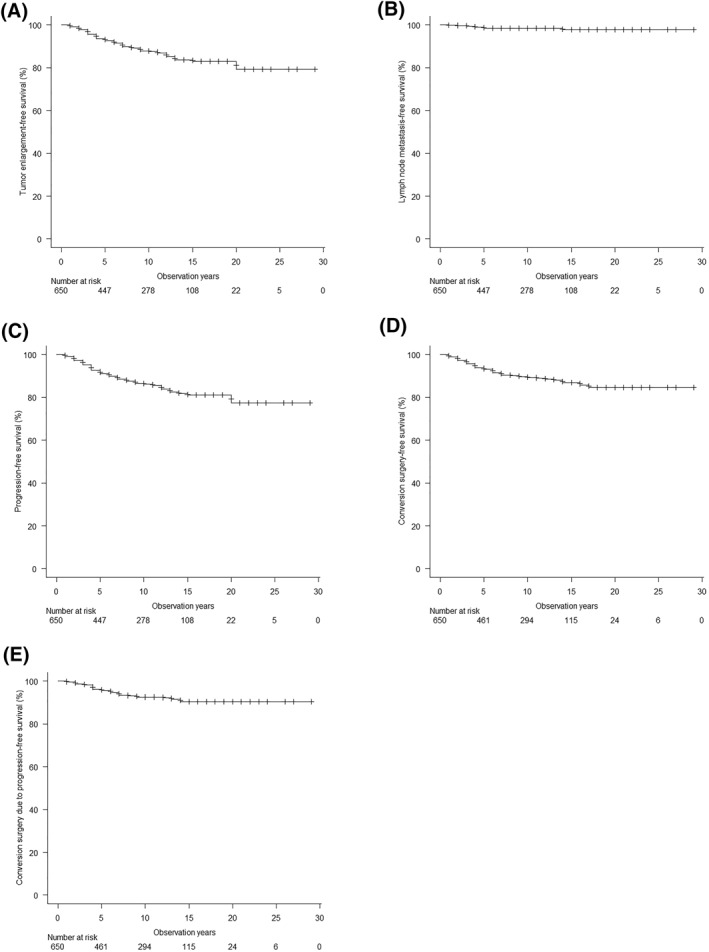
Outcomes of active surveillance for low‐risk papillary thyroid carcinoma. (A) Tumor enlargement‐free survival. (B) Clinically apparent lymph node metastasis‐free survival. (C) Progression‐free survival. (D) Conversion surgery‐free survival. (E) Conversion surgery due to progression‐free survival.

During AS, tumor enlargement occurred in 71 patients (10.9%) and 9 patients (1.4%) developed LNM. No distant metastases or cause‐specific deaths were observed, but 20 patients died from unrelated causes. Overall, 80 patients (12.3%) showed progression. Cumulative progression‐free survival rates at 10 and 20 years were 81.1% and 77.4%, respectively (Figure 2). Univariate and multivariate analyses of risk factors for progression during AS revealed younger age and weak calcification in addition to female sex as significant predictors of progression (Table 2).

**TABLE 2:** Univariate and multivariate analyses of risk factors for progression during active surveillance for low‐risk papillary thyroid carcinoma.

Risk factor	Univariate analysis			Multivariate analysis		
	Hazard ratio	95% confidence interval	*p*‐value	Hazard ratio	95% confidence interval	*p*‐value
Initial age	0.98	0.96–0.99	**0.0050**	0.98	0.96–0.99	**0.022**
Sex (female)	5.94	1.46–24.18	**0.013**	4.87	1.19–20.0	**0.028**
Initial tumor size	0.99	0.89–1.09	0.81			
Initial calcification (weak)	2.55	1.31–4.95	**0.0057**	2.13	1.10–4.16	**0.026**
Initial vascularity (rich)	1.44	0.83–2.49	0.19	1.42	0.82–2.45	0.22
Multifocality (present)	0.86	0.49–1.51	0.60			
Initial TSH	1.06	0.90–1.26	0.47	1.05	0.89–1.24	0.55
TgAb and/or TPOAb (either positive)	1.08	0.68–1.68	0.76			
Family history of thyroid cancer (present)	<0.0001	0‐inf	0.99			
History of other malignancy (present)	1.17	0.72–1.91	0.53			
Duration of follow‐up	<0.0001	0‐inf	0.98			

*Note*: TSH, thyrotropin; TgAb, anti‐thyroglobulin antibody; TPOAb, anti‐thyroid peroxidase antibody. Bold values indicate statistical significance.

Eventually, 62 patients (9.5%) underwent conversion surgery (Figure 2) due to tumor enlargement in 31 cases, LNM in 9 cases, change in patient preference in 18 cases, and other reasons in 4 cases (appearance of new PTC lesion, concern regarding extrathyroidal extension of the PTC, onset of Graves' disease, and enlargement of coexistent benign thyroid nodule in 1 case each). No recurrence has been observed in any patients who underwent conversion surgery thus far.

Timings of progression and conversion surgery were examined in terms of age and duration of follow‐up (Table 3). Median age and duration of follow‐up at the time of progression were 55 years (range, 24–84 years) and 4 years (range, 1–20 years), respectively. Five patients showed progression after reaching 80 years old and only 2 patients showed progression after 15 years of follow‐up. Median age of the 40 patients who underwent conversion surgery due to progression was 53 years (range, 27–81 years) and only 1 patient was over 80 years old. The median duration of follow‐up at the time of conversion surgery was 4 years (range, 1–14 years). Thirty‐seven conversion surgeries (92.5%) were conducted within the first 10 years and no surgery was carried out beyond 15 years of follow‐up.

**TABLE 3:** Timing of progression and conversion surgery for patients with low‐risk papillary thyroid carcinoma under active surveillance.

Age (years)	Number of patients^a^	Tumor size enlargement	Development of lymph node metastasis	Conversion surgery due to progression
a) Relationship between age and progression/conversion surgery				
39	109	11 (10.1%)	2 (1.8%)	9 (8.3%)
40–59	499	31 (6.2%)	5 (1.0%)	18 (3.6%)
60–79	530	24 (4.5%)	2 (0.4%)	12 (2.3%)
80	75	5 (6.7%)	0 (0.0%)	1 (1.3%)

Duration of follow‐up (years)	Number of patients^b^	Tumor size enlargement	Development of lymph node metastasis	Conversion surgery due to progression
b) Relationship between the duration of follow‐up and progression/conversion surgery				
1–4	650	36 (5.5%)	6 (0.9%)	27 (4.2%)
5–9	461	23 (5.0%)	2 (0.4%)	10 (2.2%)
10–14	294	10 (3.4%)	1 (0.3%)	3 (1.0%)
15	145	2 (1.4%)	0 (0.0%)	0 (0.0%)

^a^ Number of patients out of the total 650 who underwent ultrasound examinations within the age range. Patients were censored at the point of confirmed progression.

^b^ Number of patients out of the total 650 who underwent ultrasound examinations during the observation period. Patients were censored at the point of confirmed progression.

**FIGURE 3:**
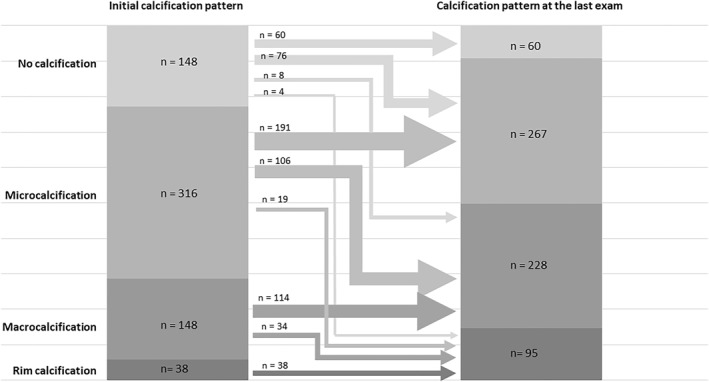
Changes in the tumor calcification pattern of low‐risk papillary thyroid carcinoma under active surveillance.

**FIGURE 4:**
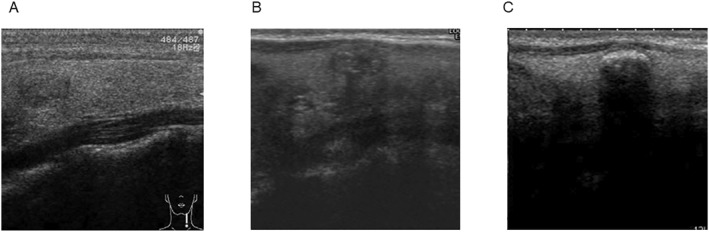
A case showing temporal enhancement of calcification during active surveillance (AS) (female, sagittal section of the left thyroid lobe). (A) At the initiation of AS in 2009 (age 29 years), tumor measured 7 × 6 × 5 mm and was accompanied by microcalcification. (B) In 2016 (age 36 years), tumor measured 6 × 6 × 5 mm and was accompanied with macrocalcification. (C) In 2023 (age 44 years), tumor measured 6 × 6 mm and was accompanied with rim calcification.

Changes in the tumor calcification pattern are shown in Figure 3. The initial calcification pattern was as follows: 148 cases (22.8%) with no calcification; 316 cases (48.6%) with microcalcification; 148 cases (22.8%) with macrocalcification; and 38 cases (5.8%) with rim calcification. However, as of the last assessment, these numbers had changed to 60 cases (9.2%), 267 cases (41.1%), 228 cases (35.1%), and 95 cases (14.6%), respectively. During AS, 247 tumors (38.0%) showed an enhancement in the calcification pattern. One example is illustrated in Figure 4. The relationships between tumor calcification pattern and patient age, duration of follow‐up, and rate of tumor size enlargement were examined (Table 4). The degree of calcification correlated significantly with older age and longer observation periods. The rate of tumor enlargement correlated inversely with the degree of tumor calcification. No progression occurred after the development of rim calcification.

**TABLE 4:** Tumor calcification pattern and progression of low‐risk papillary thyroid carcinoma under active surveillance.

Calcification at initial exam	Median age at initial exam (years)	Tumor size enlargement	Development of lymph node metastasis
No (*n* = 148)	50.5	25 (16.9%)	2 (1.4%)
Micro (*n* = 316)	54.0	39 (12.3%)	4 (1.3%)
Macro (*n* = 148)	55.0	7 (4.7%)	2 (1.4%)
Rim (*n* = 38)	59.5	0 (0.0%)	1 (2.6%)
*p*‐value	**<0.0001**	**0.0003**	0.71

Calcification at last exam	Median age at last exam (years)	Median duration of follow‐up (years)	Tumor size enlargement	Development of lymph node metastasis
No (*n* = 60)	56.5	6.0	11 (18.3%)	0 (0.0%)
Micro (*n* = 267)	60.0	7.0	42 (15.7%)	4 (1.5%)
Macro (*n* = 228)	66.0	10.0	16 (7.0%)	4 (1.8%)
Rim (*n* = 95)	70.0	11.0	2 (2.1%)	1 (1.1%)
*p*‐value	**<0.0001**	**<0.0001**	**<0.0001**	0.96

*Note*: Bold values indicate statistical significance.

**FIGURE 5:**
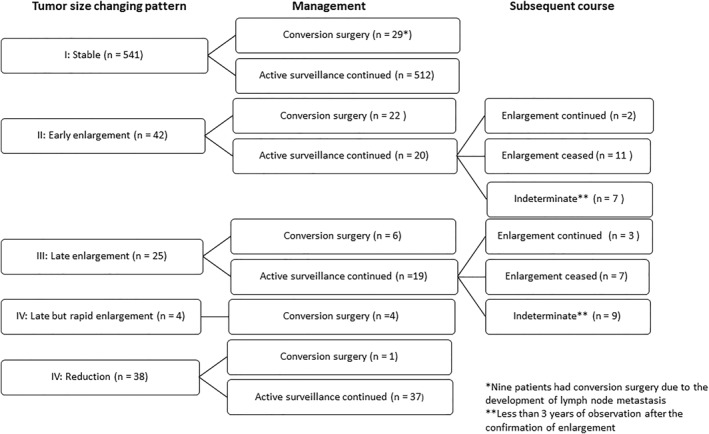
Patterns of tumor size change and subsequent courses of low‐risk papillary thyroid carcinoma under active surveillance.

Patterns of tumor size change and subsequent courses are shown in Figure 5. The majority of cases (541 patients, 83.2%) were in the stable group. Forty‐two patients (6.5%) showed early tumor enlargement within 5 years and 22 patients (52.4%) underwent surgery at that time. Among 13 patients who continued surveillance for ≥3 years, 11 (84.6%) subsequently showed a halt in tumor growth. As for the 25 patients (15.4%) with late enlargement after 5 years of observation, 6 (24.0%) underwent conversion surgery. Of the 10 patients observed for ≥3 years afterward, 7 (70%) showed cessation of enlargement. All 4 cases (0.6%) in the late but rapid enlargement group underwent surgery. Thirty‐eight cases (5.8%) exhibited tumor reduction during the observation period.

## 4 DISCUSSION

Recent publications and guidelines have advocated a “less is more” approach to PTC, aiming to address the need to avoid overtreatment of this generally indolent disease.^6^, ^17^, ^18^, ^19^ In this context, AS has become globally recognized as a management strategy for low‐risk PTC^6^, ^20^, ^21^ because research into AS, including the present study, has consistently shown favorable outcomes when certain criteria are adhered to 22, 23, 24, 25. The rate of cancer progression was generally low and no patients developed life‐threatening disease even after conversion surgery due to progression. Further, patients with AS naturally displayed a significantly lower incidence of unfavorable events related to surgery than patients who underwent immediate surgery. Even patients who underwent conversion surgery after AS showed no difference in the incidence of unfavorable events compared to patients who received immediate surgery.^26^ In addition, most studies of patient‐reported outcomes have demonstrated better physical and mental quality of life in AS groups than in surgery groups.^27^, ^28^, ^29^, ^30^, ^31^ While the impact on medical costs would vary depending on regional healthcare systems, AS may help reduce healthcare costs.^32^, ^33^ On the other hand, several issues have been highlighted as barriers to the widespread adoption of AS as a management strategy.^34^, ^35^, ^36^, ^37^ One of these is the generally recognized need for lifelong monitoring in AS.^12^ In aging populations and regions with limited healthcare resources, some resistance may be encountered from both patients and healthcare providers due to this requirement. The present study examined the actual necessity of lifelong monitoring through an analysis of AS outcomes spanning from the 1990s to the present.

In this study, younger age was identified as a significant predictor of progression, with fewer instances of progression observed in older individuals. Advanced calcification also proved to be a significant predictor of non‐progression. Regarding the timing of progression in relation to age and duration of follow‐up, a trend was observed in which fewer cases progressed with older age and longer observation periods. Among the 71 cases of progression, 66 cases (93.0%) occurred in patients under 80 years old, and 69 cases (97.2%) were observed before 15 years of follow‐up. Conversion surgeries due to progression were conducted by 80 years old in all cases except one and 92.5% were with <10 years of observation. No conversion surgery was carried out beyond 15 years of follow‐up.

Fukuoka et al. reported a higher probability of enlargement with weaker calcification, while the cumulative calcification enhancement rate at 10 years was 51.8%.^10^ This study reaffirmed the relationship between calcification and tumor enlargement risk, indicating a tendency for calcification to strengthen with age and prolonged duration of observation, and confirming that as calcification intensifies, the risk of tumor enlargement decreases. Among cases with tumors showing rim calcification during surveillance, none displayed subsequent progression.

One byproduct of research into AS has been the elucidation of the natural history of low‐risk PTC. Miyauchi et al.^38^ reported that among 169 cases of PTMC undergoing AS, the proportion of patients with growing tumors was 40% among patients ≤40 years old compared to 17% among those ≥61 years old, as calculated by the tumor volume‐doubling rate. A validation study by Yamamoto et al.^39^ using 2129 cases found that 140 patients (6.6%) exhibited moderate or rapid growth with the incidence significantly decreasing with advancing age. Conversely, tumor regression was observed in 1200 patients (56.4%) with the incidence increasing significantly with age. Regarding the clinical course of PTMC after enlargement, Ito et al.^40^ analyzed 824 low‐risk PTMCs and reported that among tumors that had once enlarged by ≥3 mm in maximal diameter, only 7.7% showed further enlargement. Such findings suggest that while low‐risk PTC may have the potential to enlarge to some extent in younger patients, this tendency generally diminishes with age. Tuttle et al.^41^ proposed categorizing patterns of change in the tumor volume for PTC under AS into six types using a cohort of 483 patients with a median follow‐up of 3.7 years: stable; early increase in volume; late increase in volume; early increase in volume followed by stability; stability followed by increase in volume; and decrease in tumor volume. In this study, patterns of change in tumor size were classified into five categories: stable; early enlargement; late enlargement; late but rapid enlargement; and reduction. A longitudinal examination over a longer period was then conducted. While 83.2% of the cases were classified as stable, among the 23 patients in the early and late enlargement groups who continued AS for ≥3 years without conversion surgery, enlargement ceased or reduction occurred in 18 cases (78.3%).

The present analysis unexpectedly revealed sex as another significant predictor of progression. The proportion of progressors was significantly lower among males with only 2 of 91 cases (2.2%), compared to 78 of 479 cases (16.3%) among females. The higher median age in males (56 years) compared to females (54 years, *p* < 0.05) may have contributed to this difference. Since other studies have reported no difference between sexes^7^ or have identified male sex as a risk factor,^42^, ^43^ further research is needed to examine the impact of sex, taking into account other confounding variables.

This study showed several limitations. The retrospective analysis of prospective registry data from two institutions indicates a need for future validation through prospective studies. In particular, the number of cases with follow‐up exceeding 15 years remains limited. In addition, further research into the mechanisms of enhancement of calcification is desirable. On the other hand, a strength of this study was the large number of cases with nearly half undergoing observation for ≥10 years. The consistent application of a protocol throughout the study ensures high data reliability.

In conclusion, it is apparent that the progression of low‐risk PTC under AS was rare in tumors with rim calcification or in older patients (≥80 years) with long‐term follow‐up (≥15 years). Given these outcomes, reducing the frequency of surveillance or potentially ending scheduled surveillance visits may be justifiable for these patients. Moreover, instances of progression halting after enlargement are not uncommon. Performing conversion surgery immediately after enlargement is likely premature.

## AUTHOR CONTRIBUTIONS

**Iwao Sugitani:** Conceptualization; data curation; formal analysis; funding acquisition; investigation; methodology; project administration; resources; validation; visualization; writing—original draft; writing‐review and editing. **Ryuta Nagaoka:** Data curation. **Marie Saito:** Data curation. **Masaomi Sen:** Data curation. **Hiroko Kazusaka:** Data curation; formal analysis; project administration; software; visualization. **Mami Matsui:** Data curation. **Takeshi Abe:** Data curation. **Ryo Ito:** Data curation. **Kazuhisa Toda:** Data curation.

## CONFLICT OF INTEREST STATEMENT

The authors declare no conflicts of interest.

## ETHICS STATEMENT

The protocol was approved by the ethics committee of Cancer Institute Hospital in 1994. Written consent for participation was obtained after agreement based on the informed decision of the patient. The institutional review board of Nippon Medical School also approved this retrospective study using the prospectively collected database in 2019.

## Data Availability

The datasets analyzed in the current study are not publicly available due to privacy concerns and ongoing research, but are available from the corresponding author upon reasonable request.
